# Incidence of Pelvic Inflammatory Disease Associated With *Mycoplasma genitalium* Infection: Evidence Synthesis of Cohort Study Data

**DOI:** 10.1093/cid/ciaa419

**Published:** 2020-07-18

**Authors:** Joanna Lewis, Paddy J Horner, Peter J White

**Affiliations:** 1 National Institute for Health Research Health Protection Research Unit in Modelling Methodology and Medical Research Council Centre for Global Infectious Disease Analysis, Imperial College London School of Public Health, London, United Kingdom; 2 Centre for Applied Statistics Courses, UCL Great Ormond Street Institute of Child Health, London, United Kingdom; 3 National Institute for Health Research Health Protection Research Unit in Evaluation of Interventions, University of Bristol, Bristol, United Kingdom; 4 Population Health Sciences, University of Bristol, Bristol, United Kingdom; 5 Modelling and Economics Unit, National Infection Service, Public Health England, London, United Kingdom

**Keywords:** *Mycoplasma genitalium*, pelvic inflammatory disease, evidence synthesis, population attributable fraction

## Abstract

We synthesized evidence from the POPI sexual-health cohort study and estimated that 4.9% (95% credible interval, .4–14.1%) of *Mycoplasma genitalium* infections in women progress to pelvic inflammatory disease versus 14.4% (5.9–24.6%) of chlamydial infections. For validation, we predicted PID rates in 4 age groups that agree well with surveillance data.

**(See the Editorial Commentary by Vazquez and Fernández on pages 2723–5.)**

Increasing evidence indicates that *Mycoplasma genitalium* (Mgen) is a sexually transmitted infection that can lead to pelvic inflammatory disease (PID) [1]. To develop optimal testing and treatment guidelines for Mgen control, it is necessary to understand its natural history and the population burden of associated disease, about which there is currently considerable uncertainty [2].

The Prevention of Pelvic Infection (POPI) Study from England [3, 4] is the only published cohort study of PID incidence in women with and without Mgen and *Chlamydia trachomatis* (Ct) infection. We analyzed data from POPI to estimate, first, the proportion of Mgen infections that were associated with progression to PID; second, the proportion of the total PID burden that was attributable to Mgen; and third, the PID rate associated with Mgen in women aged 16–44 years in England.

## METHODS

The POPI study [3, 4] was primarily a trial of chlamydia screening, recruiting female students aged 27 years or younger in London, England, 2004–2006. Women provided self-taken vaginal swabs for chlamydia testing and were randomly allocated to testing either immediately (screened group) or after 12 months’ storage (deferred screening controls). In a retrospective substudy, Mgen infection was diagnosed in women from both randomization groups using an in-house nucleic acid amplification test [4] and stored swabs from baseline and obtained by postal follow-up 11–32 months later (median, 16 months). A total of 2378 women had baseline swabs tested for Mgen, and 900 (38%) had follow-up swabs tested. Genitourinary doctors used medical records and participant questionnaires to diagnose possible PID cases in the year following enrollment. Data on PID therefore include all cases within 1 year, not only those that were ongoing at the time of follow-up. Asymptomatic cases of PID could not be identified. We used data from both arms of the trial, since there was no difference in participant management during the follow-up period.

**Figure 1.**
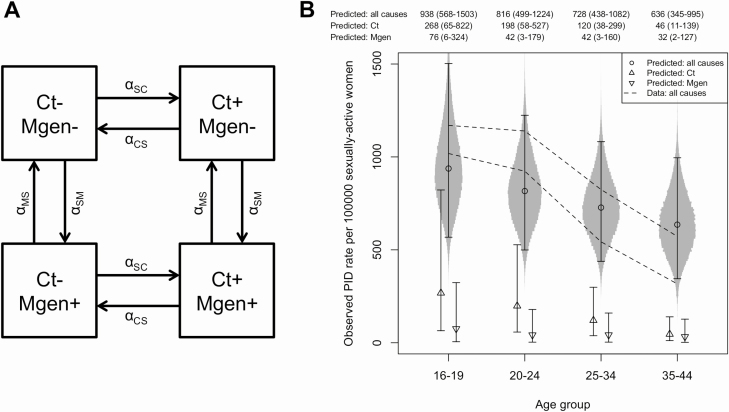
*A*, Mathematical model for Ct and Mgen infection and recovery, used to synthesize evidence. Women move between states according to a susceptible-infected-susceptible (SIS) model of natural history, with per-capita infection rates alphaSC (Ct) and alphaSM (Mgen) and recovery rates alphaCS (Ct) and alphaMS (Mgen). Neither infection affects the acquisition or recovery rate of the other. There is a “background” rate of developing PID, alphaSP, and rates attributable to Ct and Mgen infection, alphaCP and alphaMP respectively. *B*, PID rates per 100 000 women, predicted by our model using Ct and Mgen prevalence in 4 age groups in Natsal-2 (Ct) and Natsal-3 (Mgen). The prediction includes a correction to allow for the fact that only women with PID who are diagnosed (estimated to be 55% of those with symptoms; see Methods) are recorded in surveillance data. Point markers and error bars show posterior medians and 95% credible intervals, adjusted for the fraction of cases presenting for medical care; numbers are given above the plot. Circles indicate all cases, upward-pointing triangles Ct-attributable cases, and downward-pointing triangles Mgen-attributable cases. Density plots around the all-cause predictions show the sampled posterior distributions. Dashed lines indicate upper and lower bounds for PID rate observed in surveillance in 2002 reported by Price et al [5], adjusted for the proportion of women who were sexually active in each age group. Abbreviations: Ct, *Chlamydia trachomatis*; Mgen, *Mycoplasma genitalium*; Natsal, National Survey of Sexual Attitudes and Lifestyles; PID, pelvic inflammatory disease; +, positive; –, negative.

As Ct is an important cause of PID [5] we devised a mathematical model to account for PID due to both Ct and Mgen. In the model, women are in 1 of 4 states: (1) infected with neither Ct nor Mgen; (2) infected with Ct, uninfected with Mgen; (3) uninfected with Ct, infected with Mgen; or (4) infected with both Ct and Mgen. Women move between states according to a susceptible-infected-susceptible (SIS) model of natural history, with per-capita infection rates $\;\alpha_{SC}$ (Ct) and $\alpha_{SM}$ (Mgen) and recovery rates $\alpha_{CS}$ (Ct) and $\alpha_{MS}$ (Mgen). Neither infection affects the acquisition or recovery rate of the other. There is a “background” rate of developing PID, $\alpha_{SP}$, and rates attributable to Ct and Mgen infection, $\alpha_{CP}$ and $\alpha_{MP}$ respectively. The model is illustrated in Figure 1A, and full details are given in the Supplementary Information, Methods (Part 1) and Supplementary Figure 1. The prevalences of Ct and Mgen are shown by the following equations:

$$\begin{array}{l} Steady\text{-}state\;Ct\;prevalence=C^{*}=\frac{\alpha_{SC}}{\alpha_{CS}+\alpha_{SC}} \end{array}$$

$$\begin{array}{l} Steady\text{-}state\;Mgen\;prevalence=M^{*}=\frac{\alpha_{SM}}{\alpha_{MS}+\alpha_{SM}} \end{array}$$

The probability that a woman who was infected with Mgen at time zero is also infected at time *t* is:

$$\begin{array}{l} \frac{\alpha_{SM}}{\alpha_{MS}+\alpha_{SM}}+\frac{\alpha_{MS}}{\alpha_{MS}+\alpha_{SM}}e^{-\left(\alpha_{MS}+\alpha_{SM}\right)\;t} \end{array}$$

We conducted a Bayesian evidence synthesis using our model, with uninformative Gamma (1,2) priors on all parameters—except for $\alpha_{CS}$, which had a normally distributed prior, reflecting knowledge of the clearance rate of untreated Ct infection [5]. The likelihood was calculated based on 7 pieces of data from the POPI study, which are the numbers of women who were initially

1. Ct infected (137/2377);
2. Mgen infected (78/2378);
3. Mgen infected, and who were also infected when followed up, after a median time of 16 months (7/27);
4. Ct uninfected and who developed PID over the year following enrollment (31/2114);
5. Ct infected and who developed PID over the year following enrollment (7/70);
6. Mgen uninfected and who developed PID over the year following enrollment (36/2169); and
7. Mgen infected and who developed PID over the year following enrollment (3/77).

We sampled from the posterior parameter distributions using a Markov chain Monte Carlo (MCMC) algorithm, implemented in the Stan software (Stan Development Team). The code used for analysis is available at https://github.com/joanna-lewis/mgen_evidence_synthesis. We generated 75 000 samples, 15 000 from each of 5 chains, following a 5000-sample warm-up period.

For each sampled parameter set we calculated the proportion of women acquiring Mgen or Ct infection who would be expected to develop PID:

$$\begin{array}{l} \frac{\alpha_{MP}}{\alpha_{MP}+\alpha_{MS}}\;for\;Mgen\;and \end{array}$$

$$\begin{array}{l} \frac{\alpha_{CP}}{\alpha_{CP}+\alpha_{CS}}\;for\;Ct, \end{array}$$

and the proportion of PID in the POPI population that was attributable to Ct and Mgen infection:

$$\begin{array}{l} \frac{M^{*}\alpha_{MP}}{M^{*}\alpha_{MP}+C^{*}\alpha_{CP}+\alpha_{SP}}\;for\;Mgen\;and \end{array}$$

$$\begin{array}{l} \frac{C^{*}\alpha_{CP}}{M^{*}\alpha_{MP}+C^{*}\alpha_{CP}+\alpha_{SP}}\;for\;Ct. \end{array}$$

We also used our model and Ct and Mgen prevalence data from the National Survey of Sexual Attitudes and Lifestyles (Natsal) studies [6, 7] to predict PID rates in 4 age groups of women in England. As POPI recruited in 2004–2006, which was during the early stages of the roll-out (from 2003 to 2008) of England’s National Chlamydia Screening Program, we used the Natsal-2 Ct prevalence data because they reflect the situation at that time. *Mycoplasma genitalium* prevalence was estimated only by Natsal-3, but it seems likely that prevalence had remained relatively constant over the preceding years because there was little change in sexual behavior between the Natsal-2 and Natsal-3 surveys, and no specific intervention against Mgen prior to or at the time of either survey.

In POPI, only 21 of the 38 PID cases diagnosed (55%) were reported as PID by the participant or her general practitioner (GP). We therefore adjust our predicted PID rates by multiplying by a reporting fraction sampled from a Beta (22,18) distribution (mean, 55%; reflecting the observed proportion of cases reported). Following Price et al [5], we compared these adjusted predictions to 2002 PID surveillance data from genitourinary medicine (GUM) clinics, GPs, and hospital episode statistics, taking the sum of all 3 sources as an upper bound and the number in GUM clinics plus the larger of the numbers from GPs and hospitals as a lower bound. These were used to calculate PID rates per 100 000 sexually active women, taking the proportion of each age group who were sexually active from Natsal-2.

## RESULTS

We found that 4.9% (95% credible interval, .4–14.1%) of Mgen infections progress to PID, compared with 14.4% (5.9–24.6%) of Ct infections. We estimated that 9.4% (.8–28.8%) of PID in the POPI population was attributable to Mgen infection, 37.4% (14.9–63.9%) to Ct, and the remaining 51.9% (21.4–77.8%) to other causes. The posterior distributions inferred for each of the model parameters, for the percentages of each infection that progress to PID, and for the percentage of PID attributable to Mgen, Ct, and other causes, are summarized in the Supplementary Information (Supplementary Table 1; Supplementary Figures 2 and 3).

Figure 1B shows PID rates predicted by the model, based on Ct and Mgen prevalence observed in women in Natsal, in 4 age groups. After accounting for the proportion of each age group who are sexually active and the proportion of PID cases that are reported, predictions match the observed data well. The model reproduces the decrease in PID rates with age, which was due mainly to the lower Ct prevalence in older women. In younger women, more PID was attributable to Ct than to Mgen. In older age groups the amount attributable to each infection was similar, because Mgen prevalence was similar across age groups [7], whereas Ct prevalence decreased with age [8].

## DISCUSSION

We have performed the first synthesis of multiple types of data to estimate the PID burden associated with Mgen. Using data from a single study enabled us to compare the incidence of PID associated with Mgen and Ct in a self-consistent manner: we found that, in this population, the percentage of infections that progressed was substantially lower for Mgen than Ct. Our estimate of the proportion of PID in the study population that was attributable to Ct infection (37.4%; 14.9–63.9%) is comparable to other estimates made using several different methods [5], providing confidence in our method and our estimate of the proportion attributable to Mgen (9.4%; .8–28.8%).

Our estimate of 4.9% (.4–14.1%) of Mgen infections leading to PID is consistent with, and more precise than, the 7.1% (.2–33.9%) of women with incident infections who were diagnosed with PID in another observational study [9], although that study’s design only counted women with PID at the time of Mgen diagnosis and would therefore miss women whose PID episodes had ended or who would have developed PID if their infection had not been detected and treated. Our estimate is substantially lower than the 12.2% of Mgen-positive women who developed PID following termination of pregnancy [10]. This disparity is also seen with Ct, with 63% of Ct-positive women developing PID following surgical termination of pregnancy [11], compared with a 17.1% risk inferred by synthesizing evidence from several studies, which also adjusted for women with PID not presenting to care [5]. These estimates using the POPI study highlight the importance of providing young women with information on PID and access to high-quality care, as approximately 45% of women with PID were not reported in routine statistics either because of underreporting, incorrect diagnosis, or because they had symptoms associated with PID but did not visit a healthcare professional for assessment.

Compared with Ct, we have estimated that Mgen has a lower chance of progression to PID as well as a lower prevalence in young women [7], both factors that tend to reduce the cost-effectiveness of screening. Our findings support British Association for Sexual Health and HIV (BASHH) guidelines [12] advising Mgen testing in women presenting with PID but not widespread Mgen screening, as this is unlikely to be effective in reducing PID in young women and is certainly less important than Ct screening.

## Supplementary Data

Supplementary materials are available at *Clinical Infectious Diseases* online. Consisting of data provided by the authors to benefit the reader, the posted materials are not copyedited and are the sole responsibility of the authors, so questions or comments should be addressed to the corresponding author.

ciaa419_suppl_Supplementary-Material

## Notes

***Disclaimer***. The funders had no role in the study design, data collection and analysis, decision to publish, or preparation of the manuscript. The views expressed are those of the authors and not necessarily those of the Department of Health and Social Care, Department for International Development, Medical Research Council (MRC), National Health Service, National Institute for Health Research (NIHR), or Public Health England (PHE).

***Financial support.*** J. L. and P. J. W. were supported by the NIHR Health Protection Research Unit (HPRU) in Modelling Methodology at Imperial College London in partnership with PHE (grant number HPRU-2012–10080). P. J. W. was also supported by the MRC Centre for Global Infectious Disease Analysis (grant number MR/R015600/1); this award is jointly funded by the MRC and the Department for International Development (DFID) under the MRC/DFID Concordat agreement and is also part of the European & Developing Countries Clinical Trials Partnership 2 (EDCTP2) program supported by the European Union. P. J. H. was supported by the NIHR HPRU in Evaluation of Interventions at the University of Bristol in partnership with PHE (grant number HPRU-2012–10026).

***Potential conflicts of interest.*** P. J. W. reports funding from the National Institute for Health Research, outside the submitted work. P. J. H. reports payment to the University of Bristol for 2 postgraduate talks from Hologic, outside the submitted work. The other author reports no potential conflicts. All authors have submitted the ICMJE Form for Disclosure of Potential Conflicts of Interest. Conflicts that the editors consider relevant to the content of the manuscript have been disclosed.

Presented in part: International Society for Sexually Transmitted Disease Research Conference, Vancouver, Canada, 14–17 July 2019. Poster number P603.
