# Supplementary material for: Incidence of Pelvic Inflammatory Disease Associated With Mycoplasma genitalium Infection: Evidence Synthesis of Cohort Study Data
Source: Clin Infect Dis. 2020 Jul 18;71(10):2719–22. doi: 10.1093/cid/ciaa419 (PMC7744984; doi:10.1093/cid/ciaa419)
Supplement: ciaa419_suppl_Supplementary-Material [file ciaa419_suppl_supplementary-material.docx]

**Incidence of Pelvic Inflammatory Disease Associated with *Mycoplasma genitalium* Infection: Evidence Synthesis of Cohort Study Data – Supplementary Information**

**Joanna Lewis, Paddy J Horner and Peter J White**

**Contents**

1. Methods: details of the mathematical model

2. Results: posterior distributions

**1. Methods: details of the mathematical model**

We present here the full description of the mathematical model used for our analysis.

As *Chlamydia trachomatis* (Ct) is an important infectious cause of pelvic inflammatory disease (PID)^1^ we devised a model to account for PID due to Ct as well as *Mycoplasma genitalium* (Mgen). In the model women are in one of four states:

- C_1_: infected with neither Ct nor Mgen;
- C_2_: infected with Ct but uninfected with Mgen;
- C_3_: uninfected with Ct but infected with Mgen, or
- C_4_: infected with both Ct and Mgen.

Women move between states according to a susceptible-infected-susceptible (SIS) model of natural history, with forces of infection (i.e. per-capita acquisition rates)$\alpha_{SC}$ for Ct and $\alpha_{SM}$ for Mgen, and recovery rates $\alpha_{CS}$ for Ct and $\alpha_{MS}$ for Mgen. Neither infection affects the acquisition or recovery rate of the other. The model is illustrated in Figure 1A in the main text.

The system is a continuous-time Markov chain with transition matrix:

$$\left[ \begin{matrix} -(\alpha_{SC}+\alpha_{SM}) & \alpha_{SC} & \alpha_{SM} & 0 \\ \alpha_{CS} & -(\alpha_{CS}+\alpha_{SM}) & 0 & \alpha_{SM} \\ \alpha_{MS} & 0 & -(\alpha_{MS}+\alpha_{SC}) & \alpha_{SC} \\ 0 & \alpha_{MS} & \alpha_{CS} & -(\alpha_{MS}+\alpha_{CS}) \end{matrix} \right]$$

As the two infections are independent in this model, we can use standard results for a two-state Markov chain to give the steady-state prevalence of Ct and Mgen:

$$\text{Steady-state Ct prevalence}={C_{2}}^{*}+{C_{4}}^{*}=\frac{\alpha_{SC}}{\alpha_{CS}+\alpha_{SC}}$$

$$\text{Steady-state }\text{Mgen}\text{ prevalence}={C_{3}}^{*}+{C_{4}}^{*}=\frac{\alpha_{SM}}{\alpha_{MS}+\alpha_{SM}}$$

The probability that a person who was infected at time zero is also infected at time *t* is:

$\frac{\alpha_{SC}}{\alpha_{CS}+\alpha_{SC}}+ \frac{\alpha_{CS}}{\alpha_{CS}+\alpha_{SC}}e^{-\left( \alpha_{CS}+\alpha_{SC} \right) t}$ for Ct, and

$\frac{\alpha_{SM}}{\alpha_{MS}+\alpha_{SM}}+ \frac{\alpha_{MS}}{\alpha_{MS}+\alpha_{SM}}e^{-\left( \alpha_{MS}+\alpha_{SM} \right) t}$ for Mgen.

We model the cumulative number of PID cases by adding a fifth, “sink” state to the model to “record” the cumulative number of diagnosed PID cases occurring (Figure S1).


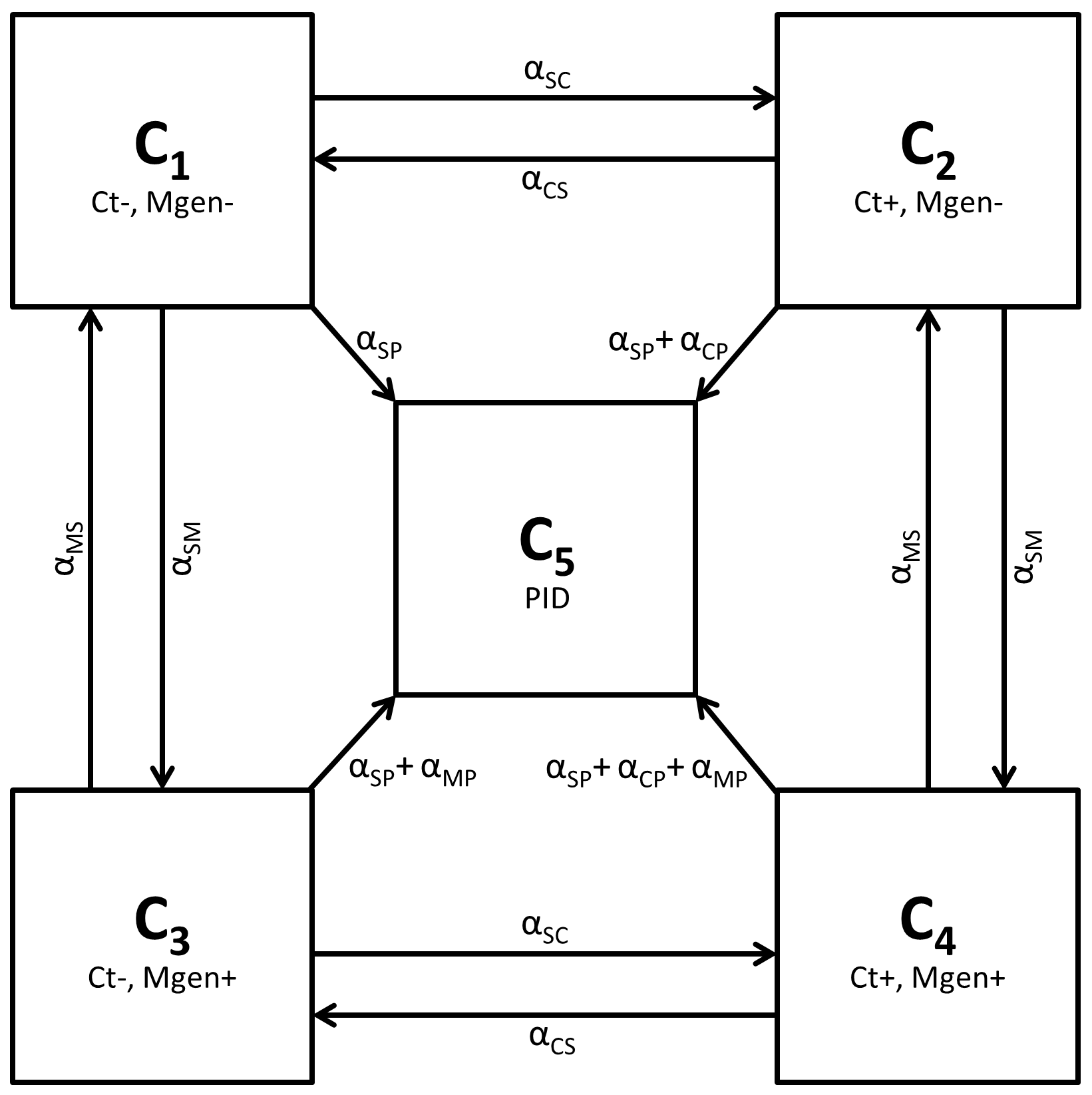


**Figure S1:** Mathematical model including a fifth compartment for PID.

This model has transition matrix:

$$\boldsymbol{Q}=\left[ \begin{matrix} -(\alpha_{SC}+\alpha_{SM}+\alpha_{SP}) & \alpha_{SC} & \alpha_{SM} & 0 & \alpha_{SP} \\ \alpha_{CS} & -(\alpha_{CS}+\alpha_{SM}+\alpha_{SP}+\alpha_{CP}) & 0 & \alpha_{SM} & \alpha_{SP}+\alpha_{CP} \\ \alpha_{MS} & 0 & -(\alpha_{MS}+\alpha_{SC}+\alpha_{SP}+\alpha_{MP}) & \alpha_{SC} & \alpha_{SP}+\alpha_{MP} \\ 0 & \alpha_{MS} & \alpha_{CS} & -(\alpha_{MS}+\alpha_{CS}+\alpha_{SP}+\alpha_{CP}+\alpha_{MP}) & \alpha_{SP}+\alpha_{CP}+\alpha_{MP} \\ 0 & 0 & 0 & 0 & 0 \end{matrix} \right]$$

where $\alpha_{SP}$is the “background” rate of progression to PID, regardless of infection state; $\alpha_{CP}$ is the rate attributable to Ct infection, and $\alpha_{MP}$ is the rate attributable to Mgen infection. Rates combine additively so that, for example, a woman infected with both Ct and Mgen would have PID progression rate $\alpha_{SP}+\alpha_{CP}+\alpha_{MP}$.

The matrix

$$\boldsymbol{Y}\left( t \right)=e^{t\boldsymbol{Q}}$$

gives the probability that an individual who was in state *i* at time 0 is in state *j* at time *t*. For example, the probability that an initially-susceptible person develops PID over follow-up period *t* is $Y_{1,5}$ and the probability that an initially-Ct-infected (but Mg-uninfected) person develops PID over follow-up *t* is $Y_{2,5}$.

Posterior parameter distributions were obtained by Markov Chain Monte Carlo sampling using the Stan software,^2^ called from the R environment.^3^ The code used for analysis is available at https://github.com/joanna-lewis/mgen_evidence_synthesis.

**2. Results: posterior distributions**

Table S1 summarizes the posterior distributions inferred for each model parameter. Figure S2 shows histograms of the sampled parameter distributions, with the corresponding priors for comparison. Figure S3 shows distributions derived from the posteriors for the proportion of Mgen and Ct infections leading to PID and the proportion of PID in the POPI population attributable to Mgen, Ct and other causes.

**Table S1:** Summary of posterior parameter distributions.

| **Parameter** | **Interpretation** | **Median (95% CrI) posterior (year^-1^)** |
| --- | --- | --- |
| α_SC_ | Force of infection (Ct) | 0.046 (0.035, 0.058) |
| α_SM_ | Force of infection (Mgen) | 0.036 (0.021, 0.062) |
| α_CS_ | Rate of clearing infection (Ct) | 0.749 (0.608, 0.888) |
| α_MS_ | Rate of clearing infection (Mgen) | 1.062 (0.645, 1.716) |
| α_SP_ | “Background” rate of developing PID | 0.010 (0.004, 0.015) |
| α_CP_ | Rate of developing PID due to Ct | 0.126 (0.047, 0.240) |
| α_MP_ | Rate of developing PID due to Mgen | 0.055 (0.004, 0.171) |

**Figure S2:** Prior (red lines) and posterior (black histograms) distributions for each of the parameters in our model. Note that uninformative priors were used, except for α_CS_.

**Figure S3:** (a) Sampled posterior distributions for the percentage of Mgen (black, thickest line) and Ct (red, medium-thickness line) infections that lead to PID. The posterior medians (95% credible intervals) were 4.9% (0.4-14.1%) for Mgen and 14.4% (5.9-24.6%) for Ct. (b) Sampled posterior distributions for the percentage of PID in the POPI population that was attributable to Mgen (black, thickest line), Ct (red, medium-thickness line) and other causes (green, thin line). Posterior medians (95% credible intervals) were 9.4% (0.7-28.6%) for Mgen, 37.4% (14.9-63.9%) for Ct, and 51.9% (21.4-77.8%) for other causes.

**References**

1. Price MJ, Ades AE, Welton NJ, Simms I, Macleod J, Horner PJ. Proportion of pelvic inflammatory disease cases caused by *Chlamydia trachomatis*: consistent picture from different methods. J Infect Dis **2016**; 214:617-24.

2. Stan Development Team. RStan: the R interface to Stan, Version 2.10.1. http://mc-stan.org/interfaces/rstan.html Accessed 7 June 2016.

3. R Core Team. R: A Language and Environment for Statistical Computing. 2016.
